# Relationship between Television Viewing and Language Delay in Toddlers: Evidence from a Korea National Cross-Sectional Survey

**DOI:** 10.1371/journal.pone.0120663

**Published:** 2015-03-18

**Authors:** Haewon Byeon, Saemi Hong

**Affiliations:** 1 Department of Speech Language Pathology & Audiology, Nambu University, Gwangju, Republic of Korea; 2 Speech-Language Pathology Center, Nambu University, Gwangju, Republic of Korea; 3 Department of Rehabilitation Medicine, Asan Medical Center, Seoul, Republic of Korea; Sun Yat-sen University, CHINA

## Abstract

**Purpose:**

This study investigated the relationship between 2-year-old children’s exposure to TV and language delay.

**Methods:**

The subjects of this study were 1,778 toddlers (906 males and 872 females) who participated in the Panel Study on Korean Children conducted in 2010. The linguistic ability of the toddlers was measured with the K-ASQ (Korean-Ages and Stages Questionnaire). The relationship between the amount of young children’s exposure to TV and language delay was analyzed with Poisson regression.

**Results:**

The average daily TV watching time of 2-year-old Korean toddlers in this study was 1.21 hours. After all confounding variables were adjusted, toddlers with over 2 hours and less than 3 hours of TV watching time had 2.7 times more risk (RR = 2.74, 95% CI: 1.13–6.65) of language delay than those with less than 1 hour of TV watching time. Those with more than 3 hours of TV watching time had approximately 3 times (RR = 3.03, 95% CI: 1.12–8.21) more risk (p<0.05). In addition, the risk of language delay increased proportionately with the increase in toddlers’ TV watching time (p = 0.004).

**Conclusion:**

Two-year-old Korean toddlers’ average daily TV watching time of more than 2 hours was related with language delay.

## Introduction

Today, TV is one of the most approachable forms of media, and 8 out of 10 households in the world possess more than one TV [1]. The TV distribution rate of Korea already surpassed 95% in the 1990s, and the digital TV distribution rate exceeded 70% in 2013 [2]. TV is known to be the most influential medium in the daily lives of contemporary people. According to a survey on 6,420 Koreans over the age of 13 conducted by the Korea Information Society Development Institute (KISDI), the average daily TV watching time of Koreans is 3.1 hours, and one in every two Koreans (46.3%) responded that TV is the most essential medium in their daily lives [2]. Especially, since TV is more approachable than other media such as computers, many toddlers worldwide spend much of their time watching TV [3–5].

Although TV is the most universal medium in homes, the relationship between TV watching and the language development of young children is still unclear. While there have been reports that TV watching has a positive effect on the linguistic and cognitive development of children [6], there have also been reports that it has a harmful effect on such cognitive abilities as attention and reading [7,8] and it has a significant relationship with language delay [9]. Moreover, there have even been reports that there is no significant correlation between TV watching time and the linguistic ability of toddlers [10]. However, these studies have been limited. For example, either the number of subjects was small or they used hospital data [3]. Second, the study on general children used a telephone survey [9]. Third, there are few epidemiological studies on the relationship between toddlers’ TV watching and communication disorders [6]. Fourth, the studies either did not examine confounding variables or only adjusted the socio-economic variables of parents [3, 9]. Fifth, few studies considered the characteristics, environmental factors, and parental factors of children with language delay.

Early childhood development is important for successful language acquisition. Linguistic development of young children proceeds continuously from birth and 5 years after birth is well-known as so-called sensitive period which is critical for language acquisition [11]. Especially, infants from 18 to 24 months old experience “word-learning explosion” in which words increase exponentially [6] and, during this period, sentences combining more than two words appear [11]. Therefore, understanding the relationship between TV watching and the language development of 2-year-old toddlers is an important subject in language development studies, and massive epidemiological studies that can represent the general population are required.

Using a nationwide representative survey of Korean toddlers, this study aimed to 1) investigate the relationship between the toddlers’ exposure to TV and language delay and 2) confirm if the risk of language delay proportionately increased with the increase in TV watching time.

## Methods

### Subjects

The source of data was the Panel Study on Korean Children (PSKC) conducted by the Korea Institute of Child Care and Education, a national policy research agency, in 2010 [12]. The PSKC has been conducted annually since 2008 in order to establish young children’s epidemiological data that can represent the Korean population. The 2010 survey was conducted on a total of 1,802 young children from June 31 to October 19 of the same year. Samples of the PSKC were collected through stratified multi-stage sampling based on 2008 resident registration population data to represent all household populations with newborn children in Korea as of 2008. The survey was approved by the IRB of the Korea Institute of Child Care and Education.

The subjects of this study were healthy young children who participated in the 2010 PSKC and were aged 24 months to 30 months. Young children diagnosed with physical disability, brain lesions, visual handicap, hearing impairment, mental retardation, autistic disorder, kidney disorder, heart disorder, respiratory disorder, liver disorder, facial disorder or epilepsy disorder from birth until October 2010 were excluded from analysis. In addition, subjects without normal development (less than −2 standard deviation) were excluded from analysis based on Children and Adolescent's Physical Growth Standard [13] by Korea Centers for Disease Control and Prevention. Among them, 24 children who did not complete the Young Children’s Development Test were ruled out, and finally 1,778 toddlers (906 males and 872 females) were selected for the analysis of this study.

### Measurement

The survey for the young children was composed of interviews with parents and the Young Children’s Development Test. In the interviews with parents, items on level of health and economic activities were surveyed with the Computer-Assisted Personal Interview (CAPI), while psychological characteristics and rearing characteristics were surveyed with the Paper and Pencil Interview (PPI) method. The CAPI was conducted by trained examiners who visited the homes of interviewees and entered data on the questionnaires in a notebook computer. PPI questionnaires were voluntarily completed by parents.

For the Young Children’s Development Test, trained examiners measured the development level of toddlers and asked their parents to complete a self-report parenting screening test. In order to enhance the accuracy of the test, examiner education was implemented three times, and quality management members checked for errors in the data surveyed.

#### Linguistic ability

The linguistic ability of the toddlers was tested using the K-ASQ (Korean-Ages and Stages Questionnaire) [14]. K-ASQ is a test adapted into the Korean language from ASQ (Ages and Stages Questionnaire) which is a standardized developmental screening test for young children at the age of 4 months to 60 months developed by Squires, Potter & Bricker [15]. The K-ASQ is composed of five sub-domains: communication, gross motor, fine motor, problem-solving, and personal–social skills. The number of questions was six per domain for a total of 30 questions. The credibility of the test (Cronbach’s α) was 0.85, and the percent agreement of concurrent inter-rater reliability was 97% [14]. In this study, language development delay was defined as cases with less than −2 standard deviation of the score of communication-domain among the K-ASQ [14].

#### TV watching time of toddlers

Average daily TV watching time of children was surveyed through parental questionnaires and classified into less than 1 hour, more than 1 hour but less than 2 hours, more than 2 hours but less than 3 hours, and more than 3 hours.

#### Confounding variables

The following confounding variables were surveyed. Main carer (parents, surrogate), household income (quartile), and size of home city (metropolitan, mid- and small-sized city, rural) were examined. Mothers’ level of education (primary school, middle school, high school, university and over), economic activities (employed, homemaker), and level of satisfaction with marriage (satisfied, moderately satisfied, dissatisfied) as well as the communication pattern between mothers and children (high, medium, low) were surveyed. Fathers’ level of education (primary school, middle school, high school, university and over) and occupation (unemployed, manual, non-manual) and the communication pattern between fathers and children (high, medium, low) were examined. Finally, toddlers’ gender, sociability (sociable, moderately sociable, non-sociable), and hospitalization experience within the past year due to disease or accident (yes, no) were surveyed.

### Statistical Analysis

The PSKC was given a weighted value so surveyed subjects represented general Koreans [12]. For the general characteristics of subjects based on the amount of exposure to TV, the weighted mean, standard error, and weighted percent were presented using technical analysis, and differences among groups were confirmed with the Rao–Scott Chi-square test.

For the relationship between TV exposure time and language delay, the risk ratio and 95% confidence interval were presented. Model 1 in the study was adjusted with environmental factors (main fosterer, household income, size of home city). Model 2 was additionally adjusted with factors related to the mother (level of education, economic activities, level of satisfaction with marriage, communication pattern with children). Model 3 was additionally adjusted with factors related to the father (level of education, occupation, communication pattern with children). Finally, Model 4 was adjusted with all confounding variables, including individual characteristics of the young children (gender, sociability, hospitalization experience within the past year due to disease or accident).

The Cochran–Armitage trend test was used to determine whether the significant probability of each category had a linear trend relationship with the reference group after the p-value was confirmed in Poisson regression. MINITAB version 13 (Minitab Inc., State College, Pennsylvania) was used for all analyses.

## Results

### General Characteristics of Subjects

The general characteristics of subjects are presented in Table 1. The average daily TV watching time of toddlers was 1.21 hours (range: 0–8, standard deviation: 0.99), and the percentage of toddlers in the “more than 1 hour but less than 2 hours” group was the highest (44.1%). Mostly, surrogates (eg. nursery teacher) were the main carers (51.8%), mothers were homemakers (67%), fathers were employed in non-manual labor (65.2%), and parents were university graduates. For communication patterns, both mothers (67.6%) and fathers (62.4%) had high levels of communication with children. The prevalence of language delay in toddlers was 5.2%.

**Table 1:** General characteristics of subjects.

Variables	weighted % (n = 1,778)
Language delay	
Yes	5.2
Average daily TV watching time	
< 1 hour	23.3
1–2 hour	44.1
2–3 hour	24.0
> 3 hour	8.6
Sex	
Male	51.3
Female	48.7
Sociability	
Sociable	82.1
Moderately sociable	14.4
Non-sociable	3.6
Hospitalization experience within the past year	
Yes	15.3
Main carer	
Parents	48.2
Surrogate	51.8
Household income	
1st quartile	24.8
2nd quartile	30.3
3rd quartile	20.8
4th quartile	24.1
Size of home city	
Metropolitan	40.2
Mid- and small-sized city	40.9
Rural	18.9
Mothers’ level of education	
≤ High school	29.0
≥ College	71.0
Mothers’ economic activities	
Employed	33.0
Homemaker	67.0
Level of satisfaction with marriage	
Satisfied	69.7
Moderately satisfied	24.5
Dissatisfied	5.8
Communication pattern between mothers and children	
High	67.6
Medium	29.3
Low	3.1
Fathers’ level of education	
≤ High school	26.0
≥ College	74.0
Fathers’ occupation	
Unemployed	9.1
Manual	65.2
Non-manual	25.7
Communication pattern between fathers and children	
High	62.4
Medium	26.5
Low	11.1

### Characteristics of Subjects Based on Language Development Level

The characteristics of subjects based on language development level are presented in Table 2. Results of the Rao–Scott Chi-square test indicated that the normal group differed significantly from the language delay group in TV watching time, gender, sociability, household income, communication pattern between mothers and toddlers, father’s level of education, and father’s occupation (p<0.05). The prevalence rate of language delay was high for the following toddlers: Those who watched TV more than 3 hours (11.0%), were male (6.9%), were non-sociable (9.9%), had little communication with their mother (12.8%), had a father who was a high school graduate or lower (7.9%), and had an unemployed father (9.6%).

**Table 2:** Characteristics of subjects based on language development level, weighted %.


Variables	Normal (n = 1,686)	LD* (n = 92)	p
Child characteristics			
Average daily TV watching time			0.001
< 1 hour	96.8	3.2	
1–2 hour	96.5	3.5	
2–3 hour	92.0	8.0	
> 3 hour	89.0	11.0	
Sex			<0.001
Male	93.1	6.9	
Female	96.7	3.3	
Sociability			0.046
Sociable	95.1	4.9	
Moderately sociable	94.2	5.8	
Non-sociable	90.1	9.9	
Hospitalization experience within the past year			0.533
No	92.9	7.1	
Yes	95.2	4.8	
Caring environments			
Main carer			0.401
Parents	94.6	5.4	
Surrogate	95.0	5.0	
Household income			0.043
1st quartile	9.24	7.6	
2nd quartile	94.3	5.7	
3rd quartile	96.6	3.4	
4th quartile	96.4	3.6	
Size of home city			0.087
Metropolitan	93.2	6.8	
Mid- and small-sized city	95.6	4.4	
Rural	96.5	3.5	
Mother characteristics			
Mothers’ level of education			0.358
≤ High school	93.3	6.7	
≥ College	95.4	4.6	
Mothers’ economic activities			0.724
Employed	94.9	5.1	
Homemaker	94.8	5.2	
Level of satisfaction with marriage			0.080
Satisfied	95.4	4.6	
Moderately satisfied	93.3	6.7	
Dissatisfied	94.9	5.1	
Communication pattern between mothers and children			<0.001
High	96.4	3.6	
Medium	91.8	8.2	
Low	87.2	12.8	
Father characteristics			
Fathers’ level of education			0.037
≤ High school	92.1	7.9	
≥ College	95.8	4.2	
Fathers’ occupation			0.005
Unemployed	90.4	9.6	
Manual	96.0	4.0	
Non-manual	93.5	6.5	
Communication pattern between fathers and children			0.123
High	95.7	4.3	
Medium	94.1	5.9	
Low	91.7	8.3	

LD: language delay

* Language delay was defined as cases with less than 2%ile of the total score of communication among the K-ASQ.

### Relationship between Toddlers’ TV Watching and Language Delay

The relationship between toddlers’ TV watching and language delay is presented in Table 3. The results of the Poisson regression analysis showed that in Model 1 in which toddlers’ environmental factors (main fosterer, household income, size of living city) were adjusted, toddlers with more than 2 hours but less than 3 hours of TV watching time had around 2.5 times (RR = 2.54, 95% CI: 1.20–5.34) more risk of language delay. Those with more than 3 hours had about 3 times (RR = 3.35, 95% CI: 1.45–7.71) more risk (p<0.05).

**Table 3:** Poisson regression analyses of the association between average daily TV watching time of children and language delay, Risk ratio and 95% CI.

	Model 1		Model 2		Model 3		Model 4	
	RR (95% CI)	p for trend	RR (95% CI)	p for trend	RR (95% CI)	p for trend	RR (95% CI)	p for trend
< 1	1	0.001	1	0.001	1	0.002	1	0.004
1–2	1.22 (0.58, 2.56)		1.32 (0.59, 3.00)		1.45 (0.61, 3.44)		1.43 (0.59, 3.45)	
2–3	2.54* (1.20, 5.34)		2.94* (1.31, 6.62)		3.06* (1.28, 7.31)		2.74* (1.13, 6.65)	
> 3	3.35* (1.45, 7.71)		3.15* (1.25, 7.94)		3.12* (1.16, 8.35)		3.03* (1.12, 8.21)	

* p<0.05

Model 1: Adjusted for environmental factors (main fosterer, household income, size of home city).

Model 2: Additionally adjusted with factors related to the mother (level of education, economic activities, level of satisfaction with marriage, communication pattern with children).

Model 3: Additionally adjusted with factors related to the father (level of education, occupation, communication pattern with children).

Model 4: Additionally adjusted with factors related to the young children (gender, sociability, hospitalization experience within the past year due to disease or accident).

In Model 2 with mothers’ factors (level of education, economic activities, level of satisfaction with marriage, communication pattern with children) additionally adjusted, toddlers with more than 2 hours but less than 3 hours of TV watching time had around 2.9 times (RR = 2.94, 95% CI: 1.31–6.62) more risk of language delay. Those with more than 3 hours had about 3.2 times (RR = 3.15, 95% CI: 1.25–7.94) more risk (p<0.05).

In Model 3, when fathers’ factors (level of education, occupation, communication pattern with children) were additionally adjusted, the TV watching time of toddlers had an independent relationship with language delay (p<0.05).

In Model 4 with all the confounding factors adjusted including characteristics of toddlers (gender, sociability, hospitalization experience), toddlers with more than 2 hours but less than 3 hours of TV watching time had around 2.7 times (RR = 2.74, 95% CI: 1.13–6.65) more risk of language delay than those with less than 1 hour. Those with more than 3 hours had about 3 times (RR = 3.03, 95% CI: 1.12–8.21) more risk (p<0.05). The result of the Cochran–Armitage trend test revealed that the risk of language delay increased proportionately with the increase in TV watching time (p = 0.004).

## Discussion

The second year of life is a sensitive period in linguistic development for successful communication. This study analyzed the relationship between the TV watching and language delay of 2- to 2-and-a-half-year-old toddlers.

In this study, 67% of young children's mothers were housewives and nursery teachers were major fosterers of 51.8% of young children. A large part of women in Korea work outside of home and hours of labor for women workers are as long as 41.7 hours per week, the longest among OECD countries [16]. In particular, women workers in their 30s with the burden of childbirth and raising of children tend to depend on nursery school for the care of their children. With decreasing hours of mother's care, communication opportunities between mothers and young children naturally declines and young children have relatively more possibility of being exposed to TV.

In this study, the average daily TV watching time of 2-year-old toddlers was 1.21 hours, and 32.6% of toddlers watched TV for more than 2 hours. This result is similar to those of overseas epidemiological studies, which reported that toddlers under 2 watch TV for 1.2 to 1.5 hours on average daily [17, 18]. Over the last 10 years, the American Academy of Pediatrics (AAP) has recommended that children under 2 watch TV less than 2 hours a day on average [19–21]. Nevertheless, this study found that one out of three 2-year-old toddlers exceeds the recommended TV watching time.

In this study, when all the confounding variables were adjusted, toddlers with more than 2 hours but less than 3 hours of TV watching time had around 2.7 times more risk of language delay than those with less than 1 hour, and those with more than 3 hours had about 3 times more risk. Moreover, the risk of language delay increased proportionately with the increase in TV watching time. Numerous studies have reported a relationship between young children’s excessive TV watching and language delay. In an epidemiological study on 8–16-month-old infants in the U.S., watching more than 1 hour of video per day had a negative association with vocabulary acquisition [9], and in a case-control study on Thai infants under 1, those who watched TV had around 6 times more risk of delay in language development than those who did not [3]. In addition, there have also been reports that young children diagnosed as language delayed watch TV earlier and more than normal children [3]. Nevertheless, the scientific mechanism concerning excessive TV watching and language development is unclear. This study suggests four possible explanations for the relationship between excessive TV watching and language delay.

First, there is the possibility that parents who think media helps the language development of children have their children with language delays watch TV for longer times. Many studies have reported that educational media have a positive effect on the vocabulary acquisition of young children [22, 23]. Parents are not only frequently exposed to advertisements citing this kind of research but also able to purchase educational media for their young children [24]. Hence, there is a possibility that parents of children with low levels of language may have had their children watch more media [9]. However, specially made educational media differ from general TV programs in terms of content, and not all TV programs necessarily help the language acquisition of young children. For example, it has been shown that educational programs such as Dora the Explorer and Arthur, which are composed of simple narrative structures and contain pauses for children to respond, are related with higher language skills but general TV programs are not [6].

Second, it may be that because of the nature of TV, it does not help enhance communication skills. Communication is a behavior in which a speaker and listener exchange information through dialogue [25]. On the contrary, the exchange of information in TV is unilateral. Thus, it is unlikely that TV watching facilitates children’s communication opportunities.

Third, there is the possibility that excessive TV watching may have a harmful effect on the early language development of infants and young children. Two years after birth, young children acquire knowledge on language structure, and combinatorial speech appears. Thus, excessive exposure to media may have a harmful effect on the acquisition of language [9, 25]. Especially, TV images with strong visual stimuli (i.e., 3.1 scene changes, 5.2 character changes, and 3.1 object changes per minute) do not help the cognitive development of children when compared with picture books [26]. There are also reports that extended exposure to adult-directed programming over a long period of time during childhood reduces cognitive skills such as executive function [27]. Furthermore, according to a recent cross-sectional study on 276 5–18-year-old children and adolescents, excessive TV watching is associated with damage of the frontopolar area, which is related to linguistic ability [28]. In addition, excessive TV watching limits play opportunities and caregiver–child interaction [3, 29].

Especially, during TV-watching, as the attention of parents and children is focused mainly on the TV screen, both the quality and quantity of parents’ utterances tend to decrease [27, 30, 31]. In addition, a study by Pempek et al. [32] on parents with young children at the age of 1, 2 and 3 reported that utterances per minute, words per minute and new words per minute which parents express to young children significantly decreased during the process of TV watching. The researchers claimed that as interaction with parents is a vital factor in language acquisition, watching TV can have negative effect on language development of young children. Similarly, longitudinal study of Zimmerman et al. [33] on infants at the age of 2 to 24 months verified that decrease of parents' language stimulation has negative effect on the language development of the infants. This kind of deprivation of communication opportunities may cause the language delays of young children. For this reason, the AAP recommends less than 2 hours of TV watching for young children under the age of 2 [34], which seems consistent with the finding that the risk of language delay of young children significantly increased with more than 2 hours of TV watching.

Fourth, there might be yet another potential factor of language delay that this study failed to take into consideration. In order to consider potential factors of language delay, this study investigated the relationship between TV watching and language delay by including the characteristics of children, factors of parents, and environmental factors as confounding variables. Nonetheless, language delay may be affected in complex ways by various causes, including psychological factors (e.g., personality), environment, attachment to parents, as well as perception [35]. Especially, although some studies have reported the possibility that different TV programs with different qualities may have different effects on language development [6, 26], this study did not examine the program types young children watch. Nevertheless, in this study on 2-year-old toddlers, the result that the risk of delay in language development proportionately increased with the increase in TV watching time implies that excessive TV watching has a negative impact on linguistic development.

This study’s strength lies in the fact that it investigated the relationship between TV watching and language delay using a national survey. However, there are four main limitations in the present study. First, although the K-ASQ used in this study is a screening test mainly used in medical institutions, it has limitation to look meticulously into language levels. Second, this study did not examine the types of TV programs young children watch. Future studies on the relationship between TV watching and linguistic development should also analyze the types of TV programs or their qualitative level. Third, there may be potential confounding factors related to language delay other than those included in this study. In particular, family history of language delay, young children's time spent with major fosterers and the age when young children were first exposed to TV were not investigated. Fourth, in this epidemiological study, as young children's time exposed to TV was surveyed through parents' questionnaires, there is a possibility that recall bias may exist. In addition, TV exposure time was collected only at home. To minimize recall bias, 24-hour observation method is required in the future.

## Conclusion

In 2-year-old toddlers in Korea, watching TV for over 2 hours daily on average was linked with language delay. For the successful language development of young children, caregivers must provide children with communication opportunities other than TV watching such as book reading or play. Longitudinal studies to explore the causal relationship between TV watching and language delay are required.
